# An innovative visual approach to the simultaneous study of two dimensions of progress in longevity: an application to French and German regions

**DOI:** 10.1186/s12963-024-00332-2

**Published:** 2024-06-13

**Authors:** Florian Bonnet, Sebastian Klüsener, France Meslé, Michael Mühlichen, Pavel Grigoriev

**Affiliations:** 1 French Institute for Demographic Studies (INED), cours des Humanités, Aubervilliers, 93300 France; 2 Federal Institute for Population Research (BiB), Wiesbaden, Germany; 3 University of Cologne, Cologne, Germany; 4 Vytautas Magnus University, Vilnius, Lithuania

## Abstract

**Background:**

Both enhancing life expectancy and decreasing inequalities in lifespan between social groups are significant goals for public policy. To date, however, methodological tools to study progress in both dimensions simultaneously have been lacking. There is also a consensus that absolute and relative inequalities in lifespan must be studied together.

**Methods:**

We introduce a novel graphical representation that combines national mortality rates with both absolute and relative measures of social inequality in mortality. To illustrate our approach, we analyze French and German data stratified by place of residence.

**Results:**

For all-age mortality, in France we find a steady pace of decline in both mortality and in regional inequalities in mortality over recent decades. In Germany, substantial progress was made in the 1990s, mostly driven by convergence between eastern and western Germany, followed by a period of slower progress. Age-specific analyses for Germany reveal a worrying divergence in regional trends at ages 35–74 in recent years, which is particularly pronounced among women.

**Conclusion:**

Our novel visual approach offers a way to simultaneously examine two dimensions of progress in longevity, and facilitates meaningful comparisons between populations, even when their current mortality rates differ. The applied methods can be easily reproduced in any country for which long-term mortality series stratified by region, or any relevant socioeconomic characteristic, are available. It is useful for both scientific analysis and policy advice.

**Supplementary Information:**

The online version contains supplementary material available at 10.1186/s12963-024-00332-2.

## Introduction

Living conditions and survival chances have strikingly improved over the last 100 years in many countries [1, 2]. But this improvement does not necessarily indicate societal progress to the advantage of all population strata and geographical areas. Developments in social and regional inequalities are very important to policymakers. Article 174 of the Treaty on the Functioning of the European Union, for example, stipulates that “the Union shall aim at reducing disparities between the levels of development of the various regions and the backwardness of the least favored regions.” In a similar vein, the United Nations Sustainable Development Goals commit member States to reducing social inequalities, “leaving no one behind” in the push to allow all to live long healthy lives.

Likewise, in the scientific study of populations there is a long-standing interest in monitoring not only trends in general mortality, but inequalities between social strata and geographical areas. Based on solid theoretical foundations such as innovation diffusion theory [3], and using standard methodological approaches in demography as well as more advanced methods such as the beta/sigma convergence framework [4], previous studies have contributed greatly to improving the understanding of causes of both social [5–12] and regional [13–18] variations in mortality.

Previous studies on health inequalities have tended to focus exclusively on change over time in measures of the dispersion of lifespans, using a broad set of indicators expressed either in absolute or relative terms. Implicitly, a decrease in the dispersion of lifespans suggests societal progress in longevity. But drawing conclusions about the dynamics of societal progress on the basis of such inequality measures alone is complex and requires caution for two main reasons. First, although absolute lifespan inequality decreases when lifespans increase over the long run [19], it is also possible for a decrease in lifespan inequality to occur with a decrease in national lifespan. For example, a few periods of “downward convergence” occurred between 1800 and 1880 in France when regions with higher lifespans were hit particularly hard by epidemics [13]; the 2003 heat wave is another recent example. Second, a heuristic rule has been suggested [20, 21] linking very low overall mortality rates and high relative inequality (“When two groups differ in their susceptibility to an outcome, the rarer the outcome, the greater the disparity in experiencing the outcome and the smaller the disparity in avoiding the outcome.”). This could explain why inequality is higher in Nordic countries than in other European countries, despite lower national mortality rates [22, 23].

In this paper, we propose a new framework to assess what we call “societal progress” in mortality: a decrease in mortality that benefits all population strata and/or regions. Our main contribution is an innovative graphical representation that combines national mortality and inequality between subgroups measured in both absolute and relative terms. In a single figure, this representation reveals improvement/deterioration in national mortality while disentangling periods of absolute and relative convergence/divergence.

We illustrate our approach with a study on France and Germany, two neighboring Western European countries with long-term mortality series stratified by place of residence. Comparisons between the two are facilitated by their similarity in terms of number of regions (95 and 96), size and structure, as well as previous efforts to harmonize the long-term time series according to their respective current territorial divisions. But the proposed approach is general and can be easily applied to any group of countries with regional time series data on mortality or any other dimension of health inequality (e.g., socioeconomic status).

It is important to note here that interpreting analyses of regional mortality data is far from straightforward. Observed regional mortality patterns reflect both variation in population composition and differences in health-related contextual characteristics such as health care, economic productivity, environment, social conditions, and culture. Furthermore, the range of these differences varies from one country to another, likely due to the diversity of social and health policies [24].

## Data and methods

French death and population counts were obtained from the French Human Mortality Database [25] for the 95 *départements* of metropolitan France (NUTS-3, the finest geographical level of the Eurostat classification) for the period 1970–2019 and by five-year age groups (0, 1–4, 5–9, …, 90–94, 95+). For Germany, we obtained death and population counts from the German federal and state statistical offices and harmonized them for 96 *Raumordnungsregionen* for the period 1992–2019. Deaths and population counts are available for the same age ranges as in France, except for the broader age groups 1–14 and 90+.

We computed standardized death rates (SDRs) for the whole population of each geographical area as well as for six broad age groups using the 2013 European Standard Population. To reduce volatility in regional statistics, we pooled death and population counts by three-year periods: thus, for example, measures for 1995 are based on the period 1994–1996. The only exception is 2019, which is based on data for just two years, 2018 and 2019.

To assess societal progress in mortality without separately examining 191 regional time series, we focus only on three sets of indicators. National SDRs provide information on improvement in mortality at the national level (first objective). With regional SDRs, the trends in standard deviation – as an absolute measure – and in the coefficient of variation – as a relative measure – indicate the extent to which this improvement is similar across regions (second objective).

We computed both weighted and non-weighted inequality measures. To ensure that our results are not affected by changes in regional population weights over time, we present our figures using only the unweighted measures. Analyses using regional population weights yielded the same conclusions (Supplementary Material B).

All calculations were performed in R version 4.0.3 [26].

## Results

**Fig. 1:**
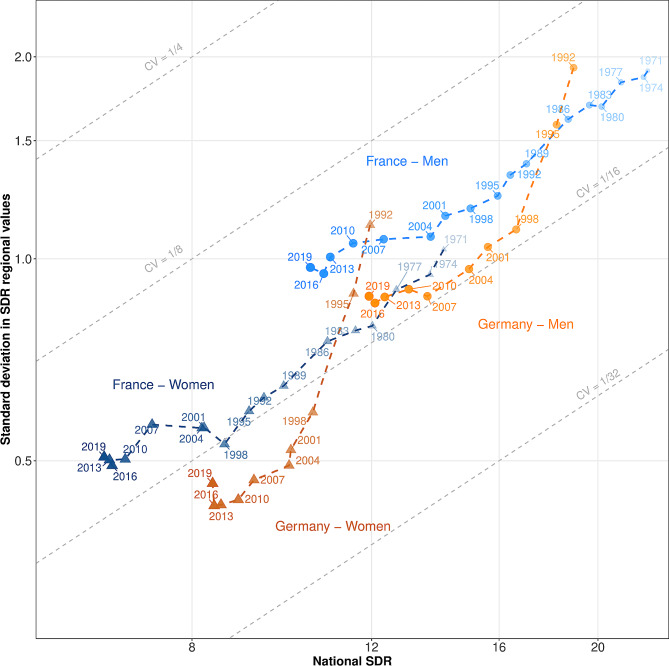
Pathways of national standardized death rates (SDRs) and regional inequalities in France (1970–2019) and Germany (1992–2019). Note: Both the x- and the y-axis values are expressed per 1,000 people with a logarithmic transformation. Societal progress in mortality occurs when the pathway moves downwards and to the left

Figure 1 presents the pathways of societal progress in mortality over time for France and Germany, differentiating men and women. Each dot describes a pair of values – the national SDR $\left(\stackrel{-}{SDR}\right)$ and the standard deviation of regional SDRs $\left(SD\right)$ – for a three-year period. The further back in time the period, the smaller and more transparent the dot.

$\stackrel{-}{SDR}$ and $SD$ are plotted on a logarithmic scale. Their relative variation across periods can be seen directly in the graph. Importantly, relative variation in the third time series – the coefficient of variation $\left(CV\right)$ – can also be seen, based on the following relationships (see Supplementary Material A for a formal demonstration):

1 $$\begin{array}{*{20}{c}}{CV = \frac{{SD}}{{\overline {SDR} }}} \end{array}$$

2 $$\begin{array}{*{20}{c}}{\frac{{\Delta CV}}{{CV}} \approx \frac{{\Delta SD}}{{SD}} - \frac{{\Delta \overline {SDR} }}{{\overline {SDR} }}} \end{array}$$

The diagonal grey lines in the graph delineate values of coefficients of variation. When the relative variation of $SD$ is higher than that of $\stackrel{-}{SDR}$, $CV$ increases; graphically, their combined pathway moves toward a higher CV line. In the opposite case, when the relative variation of $SD$ is lower than the relative variation of $\stackrel{-}{SDR}$, $CV$ decreases; graphically, the pathway moves down toward a lower CV line.

Figure 1 reveals massive progress in mortality for French women between 1971 and 1998. The dramatic fall in female mortality at the national level (39%) was accompanied by an even more rapid fall in absolute regional inequalities (49%). This difference in the pace of decline also led to a decline in relative regional inequalities: the coefficient of variation decreased by 16%, reaching 1/16. Between 1998 and 2007, women’s mortality continued to decline (with the exception of 2001–2004, due to the 2003 heat wave [27, 28]), but this progress was not accompanied by decreasing regional inequalities. Instead, the strong convergence of the previous years was succeeded by a period of both absolute and relative divergence. Since 2010, societal progress in mortality has been low, with little decline in either national mortality or regional mortality inequalities. The earlier pathway of male mortality in France shows strong similarities with that of female mortality, but for men absolute regional inequalities continued to decrease between 1998 and 2007, as did nationwide mortality between 2010 and 2019.

The German case is quite different from that of France. In 1992, just after German reunification, national mortality was at the level observed in France in the early 1980s, while regional inequalities were much higher. Societal progress in mortality was impressive for men from 1992 to 1998, and from 1992 to 2004 for women. The sharp fall in mortality (-12%) during this period was accompanied by an even greater fall in absolute regional inequalities (-47%), leading to a fall in relative regional inequalities (-44%). Thereafter, societal progress in mortality continued, but absolute regional inequality and national mortality declined at a similar pace, with almost no variation in relative regional inequalities as a result. Importantly, the pace of societal progress in mortality has decreased, and has been almost nil since 2013 for both men and women. In addition, an increase in absolute inequalities for women accompanied a stagnation in national mortality from 2016 to 2019, a combination that indicates that women’s mortality increased in some regions over this period. In particular, our analyses show an increase in women’s SDR of more than 0.25 per 1,000 in five regions. Three of them, situated in the north of the country, are almost contiguous.^1^

Figure 1 also enables comparisons between the sexes and across countries. In France, pathways in mortality over time have been more favorable for women than for men. Male mortality in 2007 was equivalent to female mortality in 1980, but absolute regional inequalities were 20% higher. In Germany, pathways have also been more favorable for women than for men in recent years. Finally, pathways were more favorable in Germany than in France during the period covered by our data, apart from the early to mid-1990s.

We also explored how the results differ if we use as mortality indicator life expectancy instead of SDR. Doing so, we produced in Figure A1 (Supplementary Material A) a figure equivalent to Fig. 1 using values of life expectancy at birth (${e}_{0}$) instead of SDR. In this case, societal progress in mortality occurs when the pathway moves downwards and to the right. Overall, the results are quite similar to those observed using SDRs. However, we observe an increase in absolute and relative inequalities since the beginning of the 21st century, both in France and in Germany. The increase is particularly marked for women.

The strong convergence between western and eastern Germany largely explains the strong societal progress in mortality in the country during the 1990s. This is illustrated by Figure A2 (Supplementary Material A), which compares the pathways for Germany as a whole to those for western and eastern Germany separately. It shows how much higher mortality rates were in the East compared to the West in 1992 (+ 16% for men, + 15% for women). The strong convergence between the SDRs of the two parts of the country largely explains the remarkable convergence observed in Germany between 1992 and 2004. Moreover, relative regional inequalities considerably decreased during this period in eastern Germany, which converged with the more favorable pathway of western Germany. Since 2004, decreasing mortality has been accompanied by constant or increasing absolute regional inequalities within the two parts of the country, resulting in a rapid increase in relative regional inequalities. Finally, regional inequalities are now higher in the West than in the East, in contrast to the early 1990s.

**Fig. 2:**
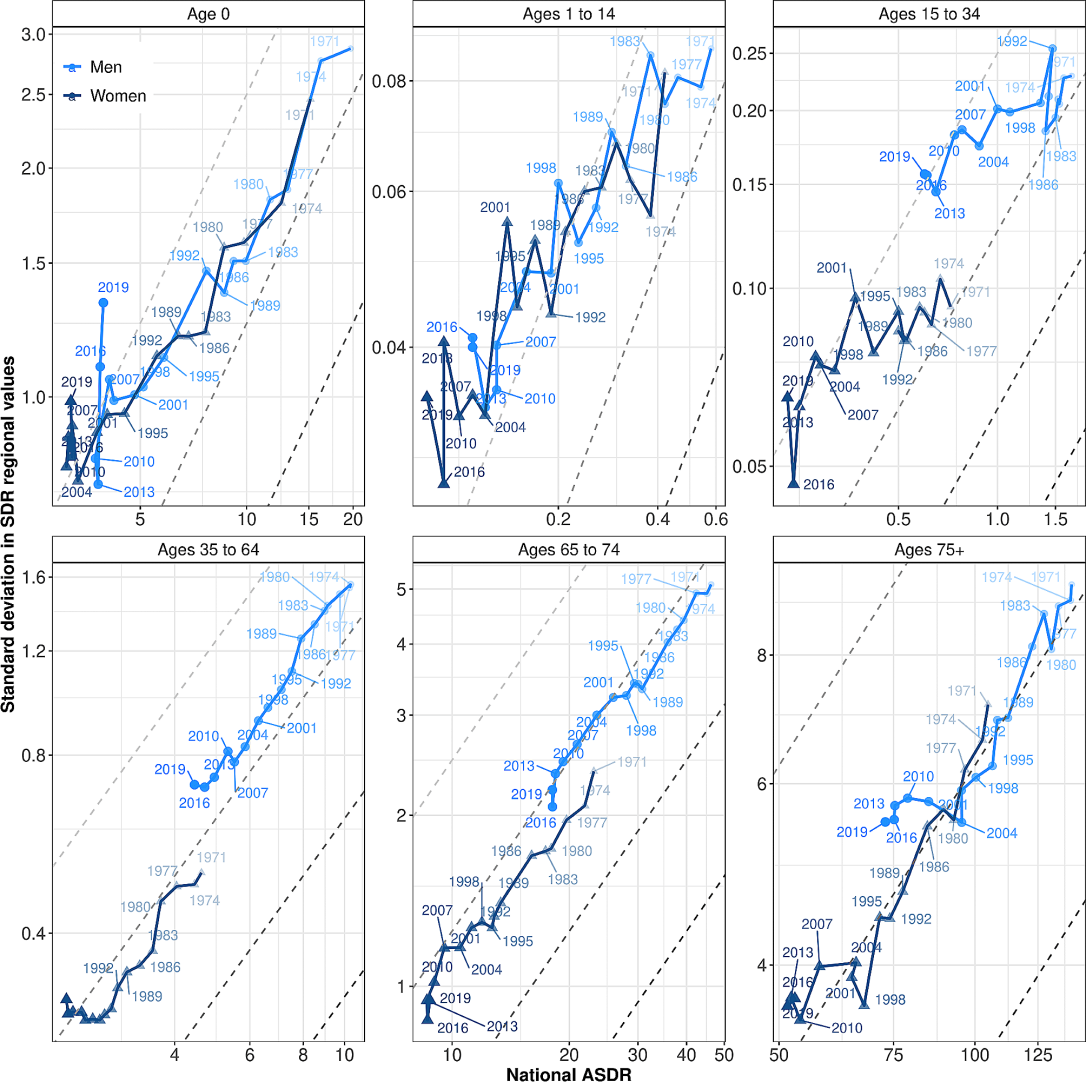
Pathways of national standardized death rates (SDRs) and regional inequalities in France for six age groups (1970–2019). Note: Both the x-axis and the y-axis values are expressed per 1,000 people with a logarithmic transformation. Societal progress in mortality occurs when the pathway moves downwards and to the left

In the next step of our analysis, we disentangle SDRs by age group. Figure 2 replicates Fig. 1 for six age groups in France. Note that we use different values transformed in a logarithmic way for the x-axis and y-axis for each panel. As in Fig. 1, values for CVs are delineated using grey dashed lines. First, the figure shows once again that mortality decline has been slowing for many age groups in recent years. This is particularly noticeable for infant mortality and for people aged 65 to 74 or above age 75. This can be likened to the increase in mortality for men aged 15 to 34 from 1985 to 1995, which was due to the AIDS epidemic. Figure 2 also reveals a strong slowdown in the absolute convergence of regional mortality rates for women aged 35 to 64 and for both men and women over age 75. This slowdown has resulted in an increase in relative regional inequalities at these ages. For men above age 75, absolute regional inequalities increased between 2004 and 2010, and have returned to levels recorded in 2001 when mortality was 25% higher. A comparison of the male and female curves shows that it was only in 2019 that male mortality at ages 35 to 64 reached a level that was surpassed by women already almost five decades earlier, in 1971. Interestingly, men’s mortality appears to have followed a path of higher relative regional inequality than women at ages above 15. A possible interpretation of this result is that regional variation in exposure to both occupational hazards and risk-taking behavior tends to be greater among men than women [29].

**Fig. 3:**
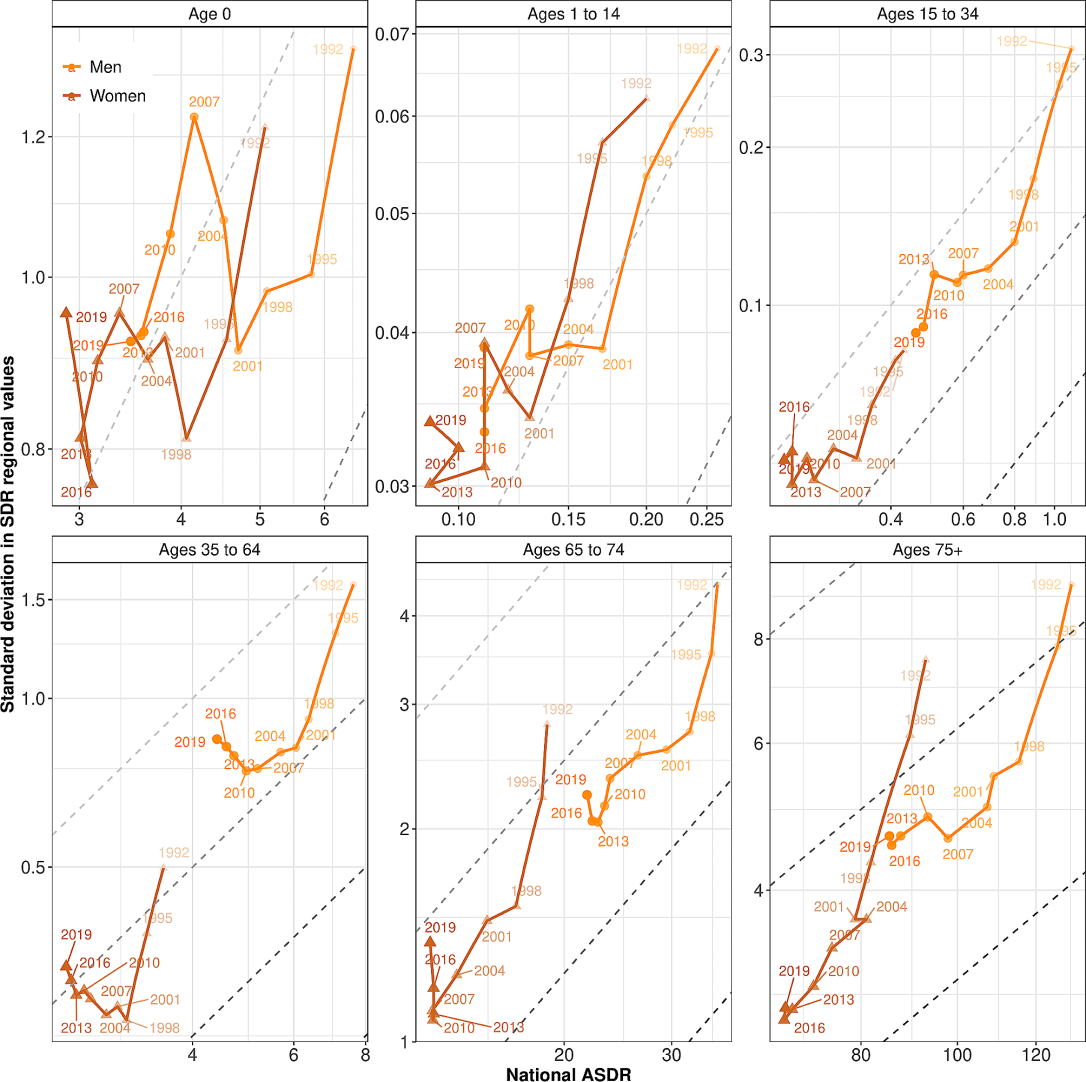
Pathways of national standardized death rates (SDRs) and regional inequalities in Germany for six age groups (1992–2019). Note: Both the x- and the y-axis values are expressed per 1,000 people with a logarithmic transformation. Societal progress in mortality occurs when the pathway moves downwards and to the left

Figure 3 presents age-specific pathways for Germany, equivalent to those for France in Fig. 2. For both men and women in age groups 35 to 64 and 65 to 74, and for women aged 15 to 34, the general picture is similar to the one seen in Fig. 1. Before 2004, declining national mortality was accompanied by even faster decline in absolute regional inequality, which resulted in a decline in relative regional inequality as well. After 2004, absolute regional inequality stalled, while mortality at the national level continued to decline, which resulted in an increase in relative regional inequality. For men aged 35 to 64 and 65 to 74 as well as for women aged 35 to 64, the low decline in mortality of recent years has been accompanied by a worrying increase in absolute regional inequalities. This “Matthew effect” [30], when decreasing national mortality is accompanied by increasing regional inequality, contrasts with the strong positive relationship we generally observe between national mortality and absolute regional inequality. Grigoriev et al. [31] argue that for women aged 35 to 64, this increase is mostly driven by regional differences in smoking: mortality rates for these ages are stalling or even increasing in some regions of central Germany. For women aged 65 to 74, the picture is even more worrying: the increase in regional inequality comes with unchanged mortality since 2007. In particular, nine regions have seen an increase in ASDR of more than 1 per 1,000: together they constitute a roughly contiguous area close to Hamburg, in the northwest of the country.^2^ Finally, the overall decline in infant mortality between 1992 and 2019 was associated with different phases of convergence (1992–1998; 2007–2019) and divergence (1998–2007).

**Fig. 4:**
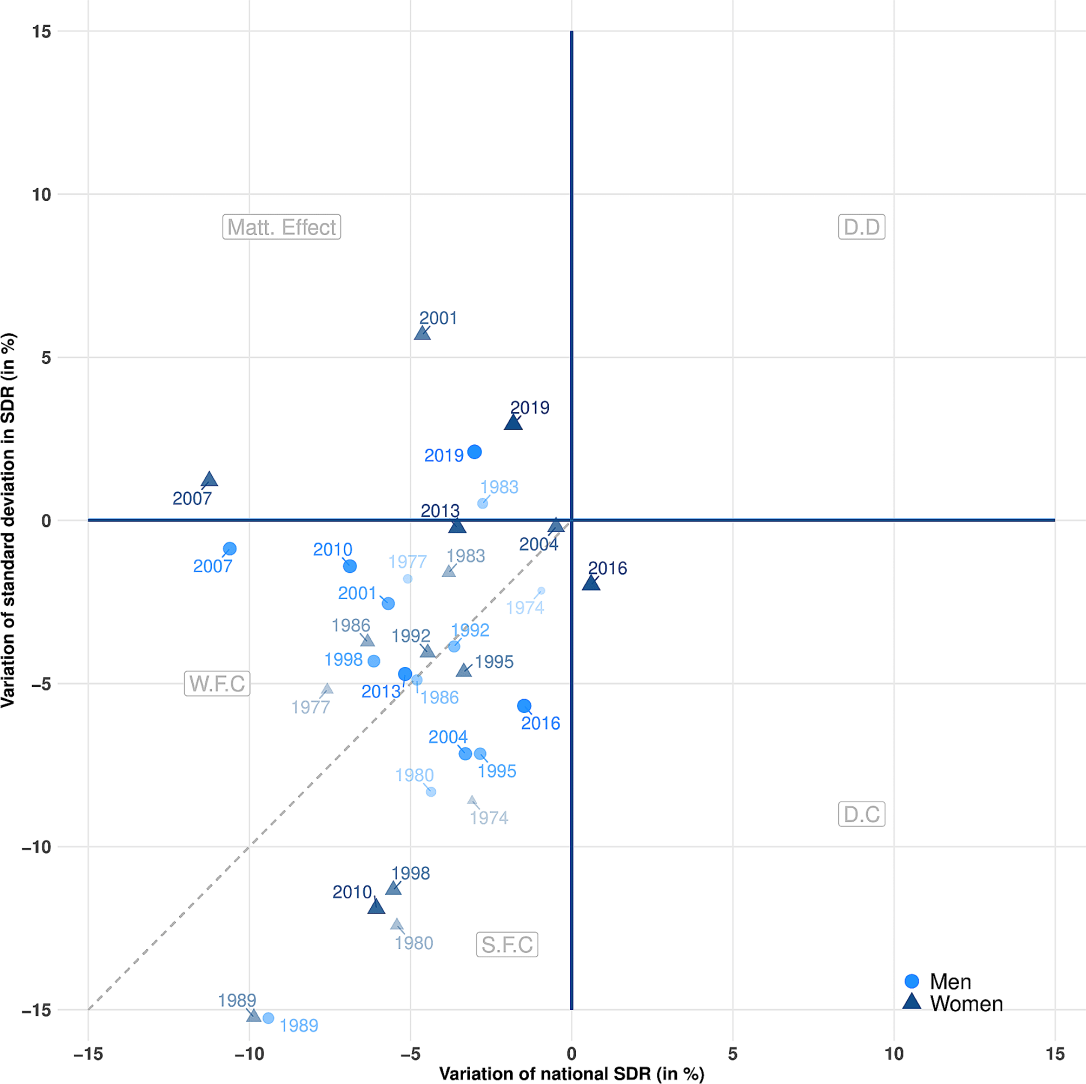
Short-term variations in societal progress in mortality in France, 1970–2019

**Fig. 5:**
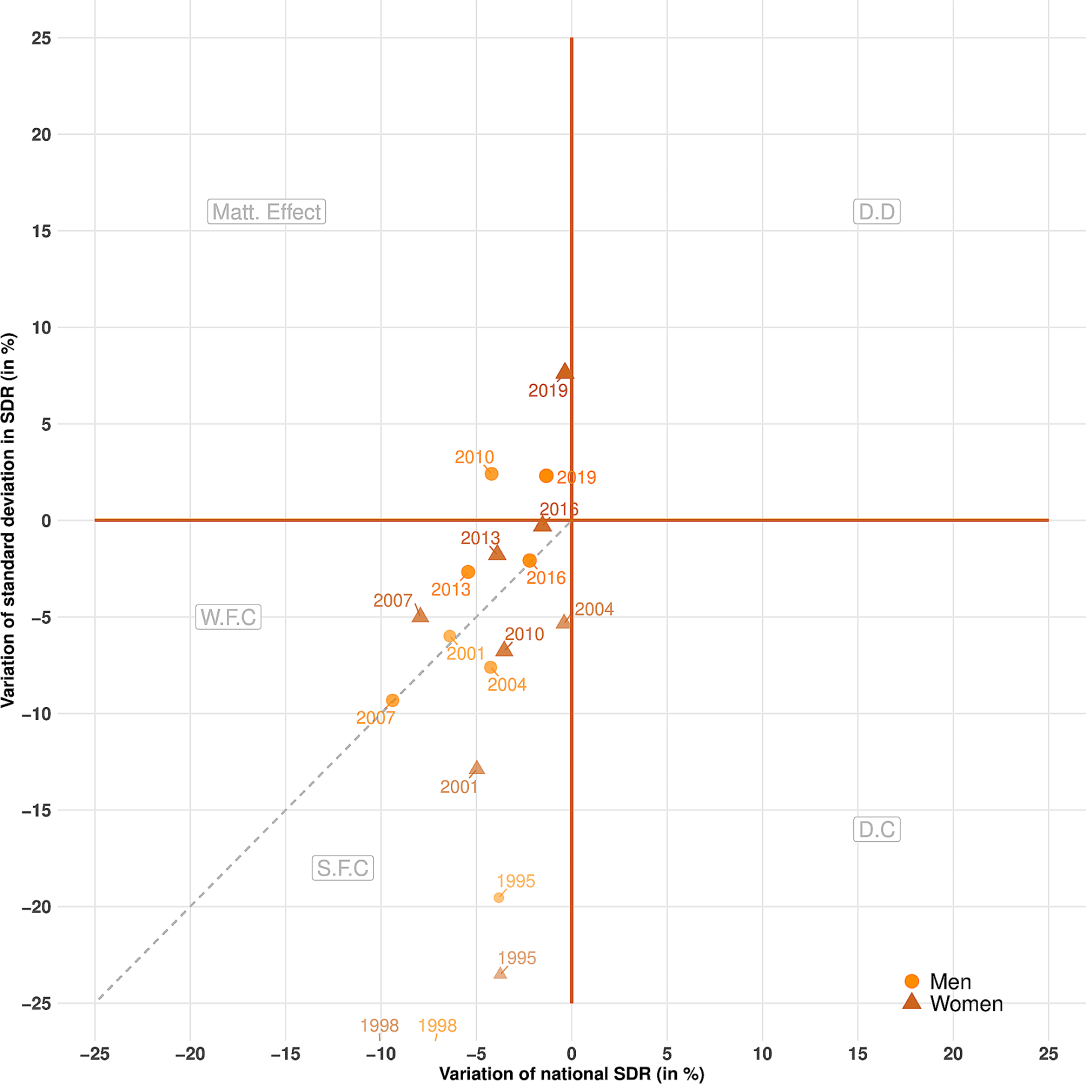
Short-term variations in societal progress in mortality in Germany, 1992–2019

While Figs. 1, 2 and 3 highlight the long-term trends in societal progress in mortality, Figs. 4 and 5 emphasize the short-term variations in progress (or its opposite) on the two objectives presented in the Data and Methods section above. Each dot plots the relative variation in national SDR (x-axis) and the standard deviation of the regional distribution of SDRs (y-axis) between two successive three-year periods for France and Germany. As an example, the dot for 1995 represents the relative variation of both national SDR and the standard deviation of regional SDRs between the successive three-year periods centered on 1992 and 1995.

Dots located on the right side of the diagram reflect periods of increasing mortality at the national level. We differentiate periods of “detrimental divergence” (DD) in the upper right, when both regional inequality and national mortality increase, from periods of “detrimental convergence” (DC) in the lower right, when regional inequality decreases but national mortality increases. Dots on the left side of the diagram, on the other hand, represent periods of decreasing mortality at the national level. We differentiate periods of a “Matthew effect” [30], in the upper left, during which progress occurs in the most favored regions, from periods of “favorable convergence” (FC), during which the progress spreads to regions that lag behind [3]. Within this quadrant, we disentangle periods of “strong favorable convergence” (SFC) and “weak favorable convergence” (WFC). In periods of SFC, below the dashed line, the decrease in absolute inequality is greater than the decrease in national mortality, leading to a decrease in relative inequality. In other words, the semi-quadrant of strong favorable convergence is the only place in the diagram that features a combined three-way decrease in overall national mortality, absolute regional inequality, and relative regional inequality.

Figure 4 presents these results for France, differentiating men and women. The further back in time the year, the smaller and more transparent the dot. Overall, most of the dots are in the lower left quadrant of the diagram, which represents societal progress in mortality. Here, both national mortality and absolute regional inequalities are decreasing. Nevertheless, a substantial proportion of these points are located outside the SFC part of the diagram, where relative regional inequality is also declining. For women, the figure shows that decreases in absolute regional inequality are decelerating: setting aside 2010, the triangles that represent changes in women’s mortality are moving toward the upper part of the diagram. Absolute inequalities even increased again between 1998 and 2001 (+ 5%), 2004 and 2007 (+ 1%), and 2016 and 2019 (+ 3%), Moreover, overall mortality increased between 2013 and 2016 (+ 1%) and then decreased only weakly between 2016 and 2019 (-2%).

Figure 5 presents the corresponding results for Germany. As revealed by Fig. 1, there was strong societal progress in mortality during the 1990s in the country. Rates of decrease in absolute regional inequalities were particularly high in 1995 and 1998 (outside of the range in the figure, between − 30% and − 40%). Overall, all the dots from before 2007 are located in the strong favorable convergence section of the figure, although the values became less favorable over time. Almost all dots from 2007 onwards are located on the opposite side of the dashed line, representing only weak favorable convergence in mortality, with increasing relative regional inequalities. Moreover, it can also be seen that the dots for more recent years have been gradually moving towards the center of the diagram: the rates of improvement in overall mortality and absolute regional inequalities are close to 0 for the periods 2013–2016 and 2016–2019. Once again, absolute inequalities increased dramatically between 2016 and 2019 for women.

## Conclusion

Here we have proposed a new visual approach to simultaneously assessing two dimensions of societal progress in longevity by combining (1) overall mortality and (2) measures of both absolute and relative regional inequality in mortality. Thanks to the mathematical properties of the chosen indicators, the approach can be used to simultaneously study both absolute and relative inequality over time.

Before discussing the implications of this visual representation, we must acknowledge its limitations. First, using this approach requires medium- or long-term data to clearly represent the pathways of societal longevity progress in each population, and to draw relevant comparisons between populations whose mortality rates are currently not at the same level. Moreover, comparisons must be made between homogeneously stratified populations: in our application, the fact that France and Germany have almost identical numbers of regions was helpful to interpret the differences between them. However, this would be less of an issue with stratification by other variables, such as level of education or income, which could also be used for this approach.

In our case study, we demonstrate how our proposed visual approach can depict the evolution of societal progress in longevity over time. In both France and Germany, the pace of societal progress in mortality has decreased, and it has fallen to nearly zero since 2013 for both men and women. More alarmingly, for both men and women aged 35 to 64 in Germany, the low rate of decline in mortality in recent years has been accompanied by increasing absolute regional inequalities in mortality. This contrasts with the strong relationship we generally observe between national mortality and absolute regional inequality. For women aged 65–74, the increase in regional inequality has even occurred in the context of unchanged mortality since the late 2000s. While mortality has continued to decrease in some regions, a large area of northwestern Germany has seen increasing mortality for more than 10 years.

We also show how this representation allows for better comparisons by sex or across countries, as differences in observed overall mortality partly explain greater or lesser absolute regional inequality. Sex-specific pathways of societal progress in mortality are more favorable in Germany than in France, as absolute regional inequalities in the former have been lower for all mortality levels with the exception of the early 1990s.

Finally, we show that results do not differ strongly whether we use standardized death rates of life expectancy at birth to evaluate regional and national levels of mortality. However, it is prudent to compare the pathways using both indicators as differences might emerge in other circumstances.

In summary, the strength of the proposed method lies in its ability to reduce the dimensionality of existing variance within countries, allowing for the simultaneous illustration of developments in both absolute and relative inequality. Moreover, this representation clearly allows better comparisons by sex or across countries than previous approaches, as it highlights how observed differences in national mortality partly explain greater or lesser absolute inequality. Thanks to its flexibility and low computational cost, our framework is well suited for use in monitoring societal progress in mortality at the international level. To this end, we provide R routines (Supplementary Material B) to replicate our graphical representations in other countries and for different types of population and regional strata.

### Electronic supplementary material

Below is the link to the electronic supplementary material.

Supplementary Material 1

## Data Availability

The raw datasets used during the current study are available from the corresponding author on reasonable request. Materials to replicate the figures are available at: https://osf.io/fj3tc/?view_only=7590c44be54846568f16f85362d37951.

## Footnotes

1 The five regions are Eastern Schleswig-Holstein, Eastern Württemberg, Saar, Southwestern Schleswig-Holstein, and Western Mecklenburg.

2 These regions are Bremen, East Frisia, Eastern Schleswig-Holstein, Hamburg-Southern Hinterland, Northern Schleswig-Holstein, Southern Schleswig-Holstein, and Southwestern Schleswig-Holstein.
