# Supplementary material for: An innovative visual approach to the simultaneous study of two dimensions of progress in longevity: an application to French and German regions
Source: Popul Health Metr. 2024 Jun 13;22:11. doi: 10.1186/s12963-024-00332-2 (PMC11177482; doi:10.1186/s12963-024-00332-2)
Supplement: Supplementary file 1 — Supplementary Material 1 [file 12963_2024_332_MOESM1_ESM.docx]

An innovative visual approach to the simultaneous study of two dimensions of progress in longevity: An application to French and German regions

# **Supplemental Material A**

**Links between national standardized death rate**$\boldsymbol{(SDR)}$**, standard deviation of regional SDRs** $\boldsymbol{(SD)}$ **and coefficient of variation of regional SDRs** $\boldsymbol{(CV)}$**.**

Let index by $t$ and $t+1$ the values in first and second periods. We can write:

$$\mathrm{CV}_{t}=\frac{\mathrm{SD}_{t}}{\bar{\mathrm{SDR}_{t}}} (1)$$

$$\mathrm{CV}_{t+1}=\frac{\mathrm{SD}_{t+1}}{\bar{\mathrm{SDR}_{t+1}}} \left( 2 \right)$$

By combining $\left( 1 \right)$ and $\left( 2 \right)$, we obtain:

$$\frac{\mathrm{CV}_{t+1}}{\mathrm{CV}_{t}}=\frac{\frac{\mathrm{SD}_{t+1}}{\mathrm{SDR}_{t+1}}}{\frac{\mathrm{SD}_{t}}{\mathrm{SDR}_{t+1}}}=\frac{\frac{\mathrm{SD}_{t+1}}{\mathrm{SD}_{t}}}{\frac{\mathrm{SDR}_{t+1}}{\mathrm{SDR}_{t}}} \left( 3 \right)$$

We call $\Delta x$ the absolute variation of $x$ between $t$ and $t+1$. Using this notation in (3), we obtain:

$$\frac{\mathrm{CV}_{t}+\Delta CV}{\mathrm{CV}_{t}}=\frac{\frac{\mathrm{SD}_{t}+\Delta SD}{\mathrm{SD}_{t}}}{\frac{\mathrm{SDR}_{t}+\Delta SDR}{\mathrm{SDR}_{t}}} (4)$$

$$1+\frac{\Delta CV}{\mathrm{CV}_{t}}=\frac{1+\frac{\Delta SD}{\mathrm{SD}_{t}}}{1+\frac{\Delta SDR}{\mathrm{SDR}_{t}}} (5)$$

We transform $(5)$using the natural logarithm and its mathematical properties:

$$ln\left( 1+\frac{\Delta CV}{\mathrm{CV}_{t}} \right)=ln\left( \frac{1+\frac{\Delta SD}{\mathrm{SD}_{t}}}{1+\frac{\Delta SDR}{\mathrm{SDR}_{t}}} \right)=ln\left( 1+\frac{\Delta SD}{\mathrm{SD}_{t}} \right)-ln\left( 1+\frac{\Delta SDR}{\mathrm{SDR}_{t}} \right) (5)$$

When $x$ is small, $ln\left( 1+x \right)\approx x$. Using this approximation in $(5)$, we finally obtain:

$$\frac{\Delta CV}{\mathrm{CV}_{t}}\approx\frac{\Delta SD}{\mathrm{SD}_{t}}-\frac{\Delta SDR}{\mathrm{SDR}_{t}} \left( 6 \right)$$

In few words, the relative variation of CV is equal to the difference between the relative variation of SD minus the relative variation of SDR.

**Additional figures**


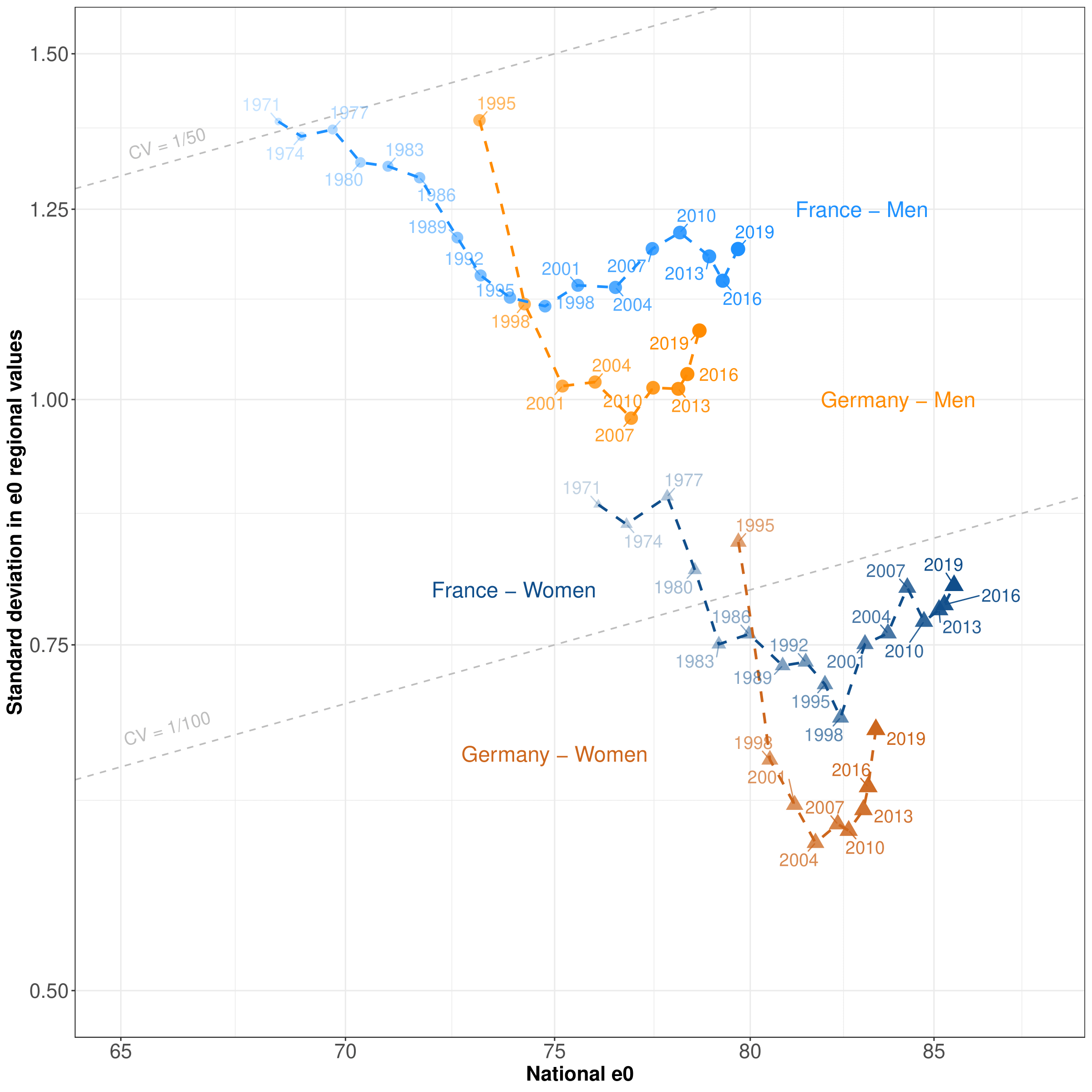


**Figure A1 :** Pathways of national life expectancy at birth (e0) and regional inequalities in France (1970–2019) and Germany (1995–2019).

Note: Societal progress in mortality occurs when the pathway moves downwards and to the right.


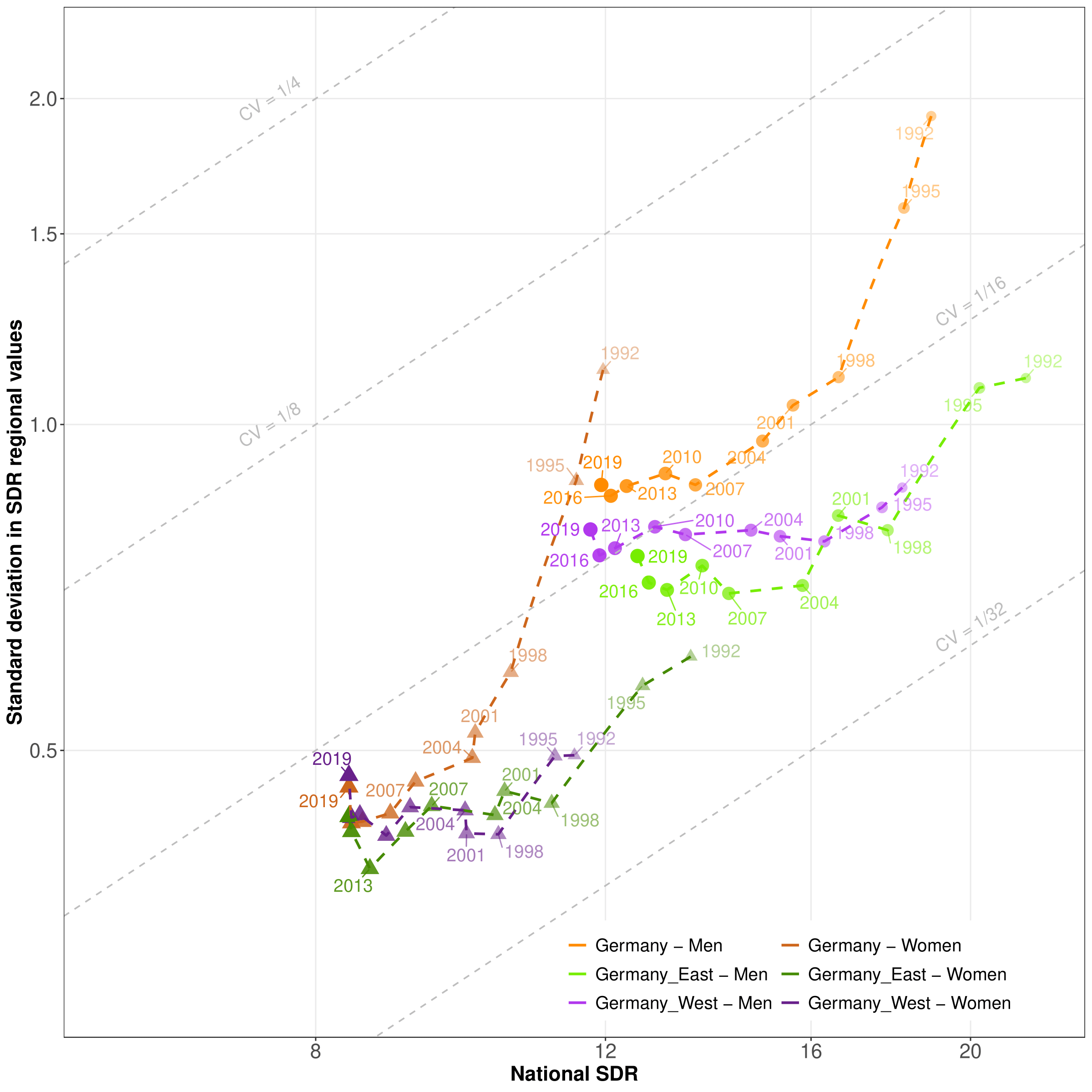


**Figure A2 :** Pathways of overall SDRs and regional inequalities in western, eastern and total Germany, 1992–2020.

Note: Societal progress in mortality occurs when the pathway moves downwards and to the left.

# **Supplemental Material B**

Data and R code to replicate our figures are available at:

<https://osf.io/h68wz/?view_only=47353f6f4b2e41cab3c761246e59d615>

Please read first “Online Supplementary Appendix.pdf”.
